# A machine learning approach to predict quality of life changes in patients with Parkinson's Disease

**DOI:** 10.1002/acn3.51577

**Published:** 2023-02-07

**Authors:** Tyler D. Alexander, Chandrasekhar Nataraj, Chengyuan Wu

**Affiliations:** ^1^ Department of Neurological Surgery Thomas Jefferson University Hospitals Philadelphia Pennsylvania 19107 USA; ^2^ Villanova Center for Analytics of Dynamic Systems (VCADS) Villanova University Villanova Pennsylvania 19085 USA

## Abstract

**Objective:**

Parkinson disease (PD) is a progressive neurodegenerative disorder with an annual incidence of approximately 0.1%. While primarily considered a motor disorder, increasing emphasis is being placed on its non‐motor features. Both manifestations of the disease affect quality of life (QoL), which is captured in part II of the Unified Parkinson's Disease Rating Scale (UPDRS‐II). While useful in the management of patients, it remains challenging to predict how QoL will change over time in PD. The goal of this work is to explore the feasibility of a machine learning algorithm to predict QoL changes in PD patients.

**Methods:**

In this retrospective cohort study, patients with at least 12 months of follow‐up were identified from the Parkinson's Progression Markers Initiative database (*N* = 630) and divided into two groups: those with and without clinically significant worsening in UPDRS‐II (*n* = 404 and *n* = 226, respectively). We developed an artificial neural network using only UPDRS‐II scores, to predict whether a patient would clinically worsen or not at 12 months from follow‐up.

**Results:**

Using UPDRS‐II at baseline, at 2 months, and at 4 months, the algorithm achieved 90% specificity and 56% sensitivity.

**Interpretation:**

A learning model has the potential to rule in patients who may exhibit clinically significant worsening in QoL at 12 months. These patients may require further testing and increased focus.

## Introduction

Parkinson disease (PD) is a progressive neurodegenerative disorder with an estimated incidence as high as 18.6 cases per 100,000 people per year.^1^ While traditionally considered a motor disorder, increasing emphasis is being placed on non‐motor features of the disease.^2^, ^3^ Both motor and non‐motor aspects of PD greatly affect the quality of life (QoL) of afflicted patients.^4^, ^5^, ^6^, ^7^ Non‐motor QoL metrics such as speech, swallowing, dressing, hygiene, and sleep are captured in part II of the Unified Parkinson's Disease Rating Scale (UPDRS‐II) while motor features are captured in the part III (UPDRS‐III) of the same survey. These scoring systems have been used across clinical studies as a validated measure of sequelae of PD symptoms.^8^, ^9^ Understanding and predicting PD progression is a major focus of research because of its importance in patient management. Yet, making such predictions about how a patient's symptoms will change over time is challenging. And while much work has been focused on this area of predicting progression from a motor standpoint, little emphasis has been placed on predicting QoL changes over time.

Historically, predictions of PD progression have focused on how motor symptoms change over time as measured by UPDRS‐III. It has been shown that different variables including sex, alcohol abuse, among others can be used to predict these UPDRS‐III changes over time.^10^, ^11^, ^12^, ^13^ Furthermore, since motor progression has been shown to be PD‐subtype‐dependent much work has emphasized early diagnosis and identification of clinical subtypes of the disease.^14^, ^15^, ^16^ Machine learning techniques have been applied to predict changes in motor symptoms over time as well as to group patients into clinical subtypes––mainly through neural network‐based classifiers and clustering algorithms, respectively.^17^, ^18^, ^19^ Prediction of QoL changes, however, have been less‐well‐emphasized. Work in predicting progression using UPDRS‐II has tended to combine those scores with a to predict motor changes as opposed to predicting changes in QoL.^19^, ^20^ Studies which have predicted QoL changes through UPDRS‐II have used various predictors including the effect of dance, deep brain stimulation, and PD drugs.^21^, ^22^, ^23^ These studies, however, have not used machine learning techniques. Furthermore, their predictions were used to evaluate the effect of interventions on QoL changes––not to measure, through simple surveys like UPDRS‐II, how QoL changes over time. No study, to our knowledge, has used UPDRS‐II scores taken over time to predict QoL changes. The goal of this study was to determine if a machine learning model could be used to predict whether or not a patient would exhibit clinically significant worsening in UPDRS‐II scores at 1 year from baseline, predicted solely from prior UPDRS‐II scores.

This study utilizes longitudinally captured UPDRS‐II scores taken from a public PD database, to predict whether a patient will exhibit clinically significant worsening in QoL within 2 years.^24^, ^25^ This technique is particularly appealing because UPDRS‐II is an easily administered survey that does not require invasive or expensive interventions. A more robust understanding of how QoL changes over time is essential for improving the treatment of patients with PD. One of the most important changes a patient and provider may need to know is whether a patient will exhibit clinical worsening in QoL over time. This knowledge will impact not only know the provider plans to treat a patient with PD, but also how a patient with PD will manage their disease. As there is currently no established method to predict whether a patient will exhibit clinically significant worsening in QoL over time, providers deal with increased uncertainty when managing patients with PD. Since machine learning algorithms have been applied to other PD prediction problems, it is logical to ask whether or not these tools can be applied to predicting QoL changes over time. Furthermore, given that UPDRS‐II is a cost‐effective and noninvasive measurement of PD sequelae, it is of interest to know whether this tool can be combined with machine learning algorithms to develop a better understanding of QoL changes in PD patients over time. This study therefore hypothesizes that machine learning techniques can be used to analyze UPDRS‐II scores over time and predict whether PD patients will exhibit clinically significant worsening at 2 years from baseline.

## Methods

### Participants

We conducted a retrospective cohort study utilizing data from the Parkinson's Progression Markers Initiative (PPMI) database (https://www.ppmi‐info.org/data). The PPMI database is a large observational study with biological samples, imaging data, well as clinical and behavioral data on PD as well as control patients. Eligibility, inclusion criteria, and exclusion criteria for the PPMI study have been previously described,^26^ but briefly, PD patients were included in the study if they had a diagnosis of PD and were at least 30 years old at the time of diagnosis. Patients were excluded if they had already initiated treatment for their PD within 60 days of study screening.

**Figure 1:**
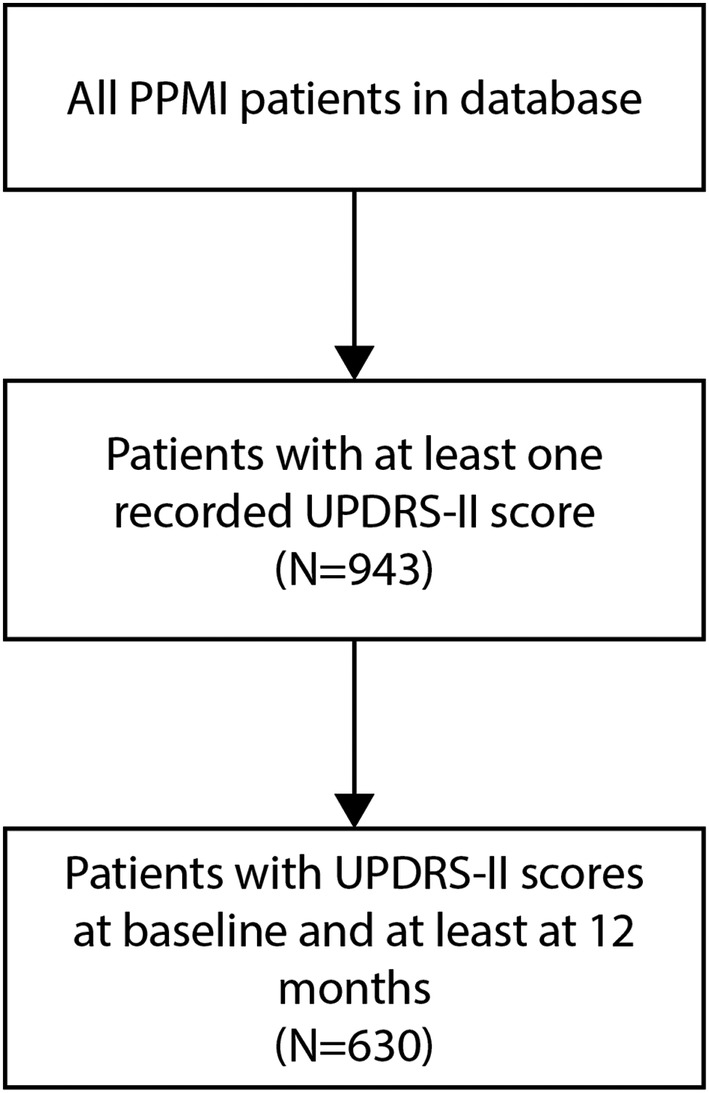
Consort diagram showing selection process for patients (*N* = 630) in the study.

In the PPMI database, composite UPDRS‐II scores were recorded at 1‐month intervals from baseline, up until a maximum of 16 months after baseline. Sub scores were not recorded nor were they used in this analysis. For the current study, patients were excluded if they did not have a UPDRS‐II score at baseline and at least at 12 months of follow‐up (Fig. 1).

### Variables

UPDRS‐II scores were obtained from the PPMI database and were treated numerically. Missing UPDRS‐II scores at any month were linearly interpolated based on the average of nearest available scores at earlier and later months. This method of linear interpolation was selected as it was validated based on general score‐change trends observed from patients with two or fewer missing scores. Change in UPDRS‐II at 12 months was calculated by taking the difference between UPDRS‐II at 12 months and UPDRS‐II at baseline. If UPDRS‐II at 12 months was more than three points greater than UPDRS‐II at baseline, that patient was considered to have clinically significant worsening. This threshold is in accordance with previous studies which have examined the minimally clinically important difference in UPDRS‐II.^27^, ^28^ Whether or not a patient had clinically significant worsening was treated as a binary variable.

### Statistical methods

Data were analyzed using a deep learning toolbox for MATLAB (The MathWorks Inc., Version 9.9.0). Confusion matrices and receiver operating characteristic (ROC) curves were generated for training, validation, and test sets of every model and used for points of comparison.

### Model development

**Figure 2:**
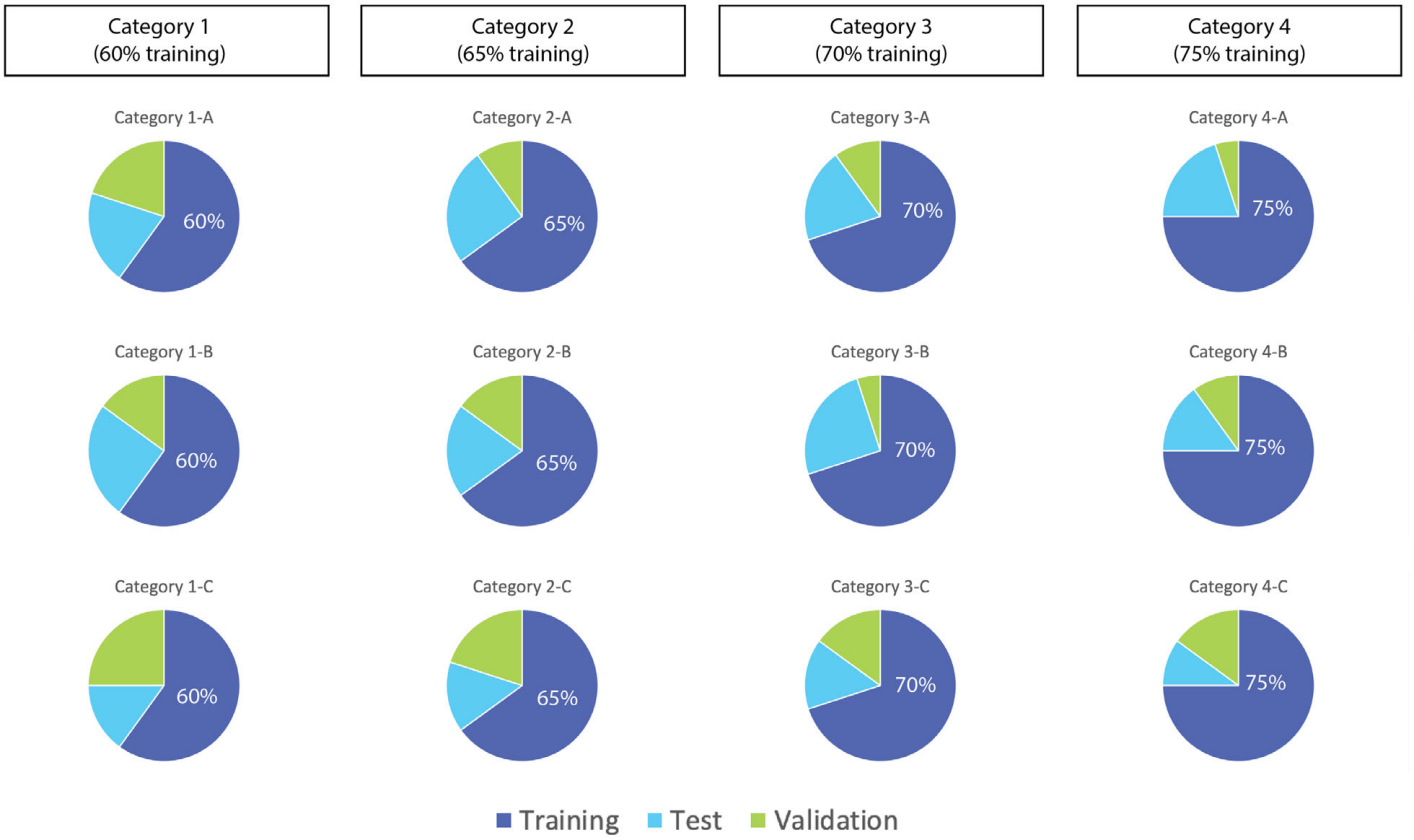
Four major categories of data divided on different ratios of training data (60% training, 65%, 70%, and 75%)––with each major category containing three unique ratios of training, testing, and validation data.

**Figure 3:**
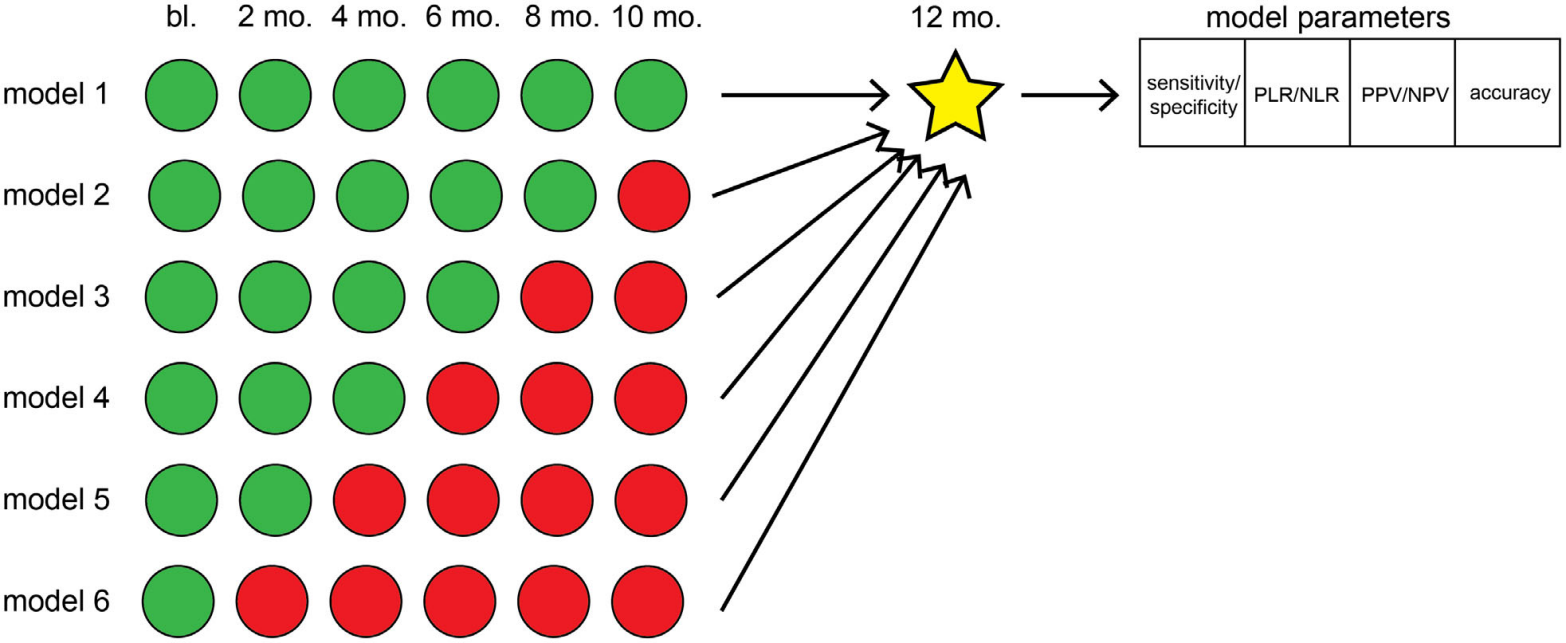
After the appropriate proportions of data usage were determined, each category was tested based on a differing number of predictors––starting at using data from 10 months to predict UPDRS‐II worsening at 12 months––with subsequent layers being peeled back.

We created a shallow artificial neural network to predict clinically significant changes in QoL at 1 year. The model was trained using a sigmoidal activation function with 10 hidden neurons. To develop the proposed model, four broad categories of data divisions were initially created––based on differing proportions of the overall data used for training. One broad category used 60% of the data for training, a second used 65% for training, a third used 70%, and a fourth used 75% of data for training. Within each category, subcategories were created where different proportions of data were used for testing and validation. For instance, in one category, 60% of the data were used for training and then various subcategories were created where testing and validation data were divided 20% and 20% in one subcategory, and 15% and 25% in another, respectively. At the end of data division and subcategory generation, 12 overall unique divisions of data existed––labeled Category 1‐A through Category 4‐C (Fig. 2). For each subdivision of data, six unique models were developed. Each model predicted UPDRS‐II score worsening at 12 months. Parameters for each model were analyzed, including sensitivity, specificity, positive likelihood ratio (PLR), negative likelihood ratio (NLR), positive predictive value (PPV), negative predictive value (NPV), and overall model accuracy. Model 1 for each subcategory used UPDRS‐II scores from baseline up to 10 months to predict clinically significant worsening. Model 2 used UPDRS‐II scores up to 8 months, and so on––until model six used only baseline UPDRS‐II to predict worsening at 12 months (Fig. 3). Because there were 12 divisions of data and 6 models for each division of data, overall, 72 different models were developed. Those models were all compared based on aforementioned extracted model parameters.

The proposed model was selected based on a balance of clinical utility and model prediction accuracy. Clinical usefulness was defined by the amount of data (i.e., months from baseline) the model required in order to accurately predict clinical worsening––where a model which requires less data were considered more clinically useful. After selecting the proposed model, the number of hidden neurons were tested within the range of two and 20, to balance model accuracy while preventing overfitting. For each variation in hidden neuron number, models were compared against one another based on the previously used metrics (ROC, confusion matrices, sensitivity and specificity, and accuracy). After this step, the proposed model was finalized.

Of note, attempts to use additional clinically relevant imaging variables in conjunction with UPDRS‐II as predictors were abandoned largely because those other variables, such as structural volumes, did not have sufficient data that could be coupled with UPDRS‐II. Using gray matter volume, for instance, would have eliminated approximately 80% of patients and as such, the only clinical predictors used were UPDRS‐II scores.

## Results

### Participants

Within the PPMI database, 943 patients who had at least one UPDRS‐II score recorded. A majority of patients with UPDRS‐II scores available (*N* = 941, 99.8%) had at least one missing score at any month. A total of 630 patients (66.8%) had UPDRS‐II scores available at baseline and at least at 12 months follow‐up. The criteria used to characterize UPDRS‐II scores as worsening or not at 12 months resulted in 35.8% of the remaining patients (*n* = 226) categorized as clinically worse and the remaining 64.1% (*n* = 404) as staying the same or improving (Table 1).

**Table 1:** Division of patients based on worsening or better/same at 12 months.

Classification, *n* (%)	All patients (*N* = 630)
Get worse (Δ UPDRS‐II >3)	226 (36%)
Stay the same/better (ΔUPDRS‐II≤3)	404 (64%)

*n* = number of patients; ΔUPDRS‐II = change in UPDRS‐II from baseline to 12 months.

### Proposed model

**Figure 4:**
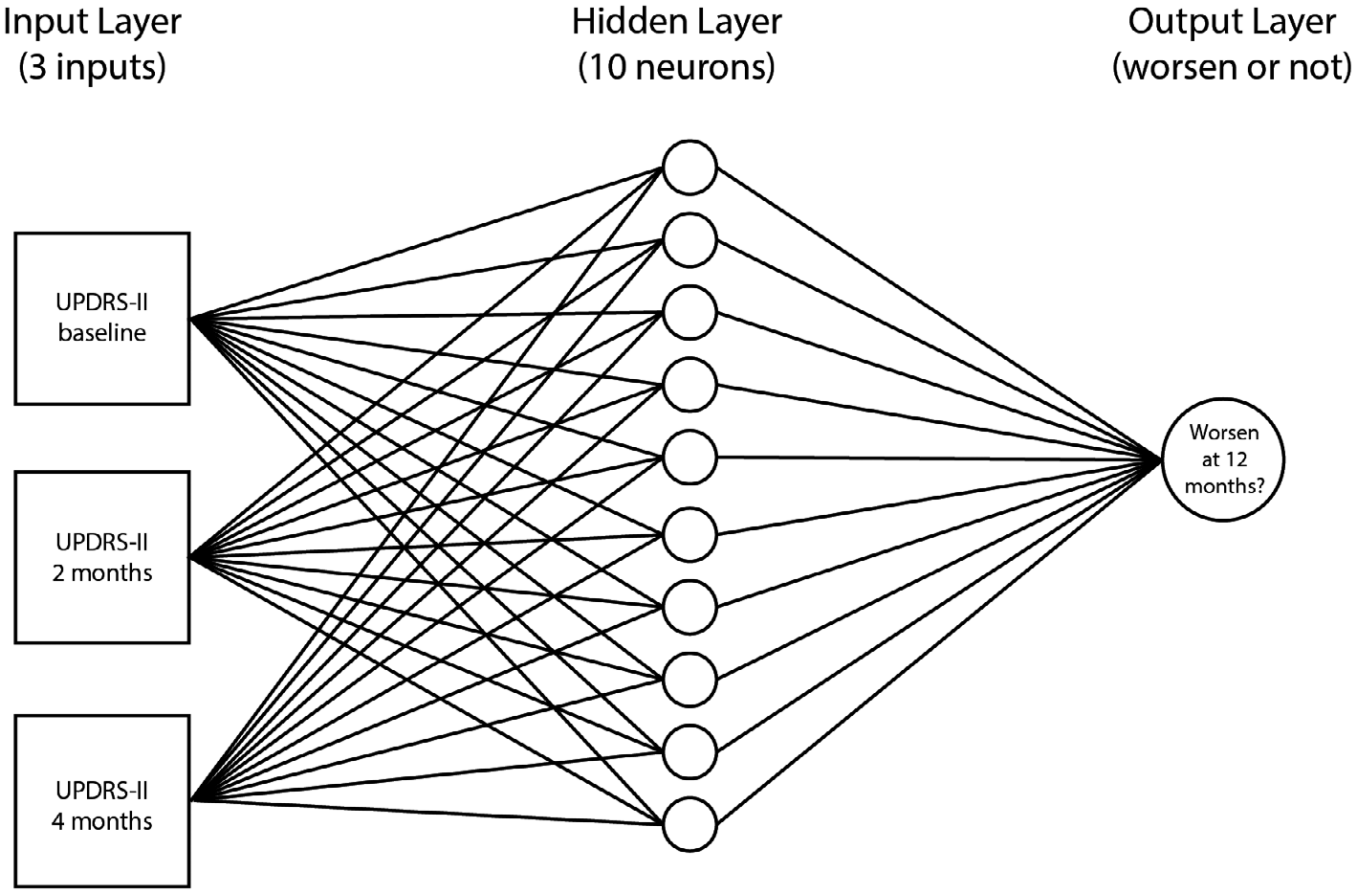
Schematic representation of the final model of the shallow neural network used to classify UPDRS‐II scores at 12 months.

The proposed model used UPDRS‐II from baseline, 2 months, and 4 months as input to predict clinical worsening at 12 months (Fig. 4). Following baseline model comparisons, the percentage of training, validation, and test data for the proposed model were 70% (*n* = 440), 15% (*n* = 95), and 15% (*n* = 95), respectively. Models which used 65% and 60% of data used for training yielded poorer performance in both sensitivity and specificity of the model. The model with 75% of data used for training showed similar accuracy to the model with 70% for training. For the final proposed model, as the number of hidden neurons was varied away from 10, the model demonstrated worse performance; with fewer hidden neurons, the model accuracy decreased. The models with more hidden neurons than 10 did not demonstrate increasing accuracy; as such, that range of hidden neurons above 10 was discarded to prevent overfitting. The final model was therefore created with 10 hidden neurons, trained on 70% of the data, and was based on predictors consisting of UPDRS‐II at baseline, 2 and 4 months.

### Model accuracy

**Figure 5:**
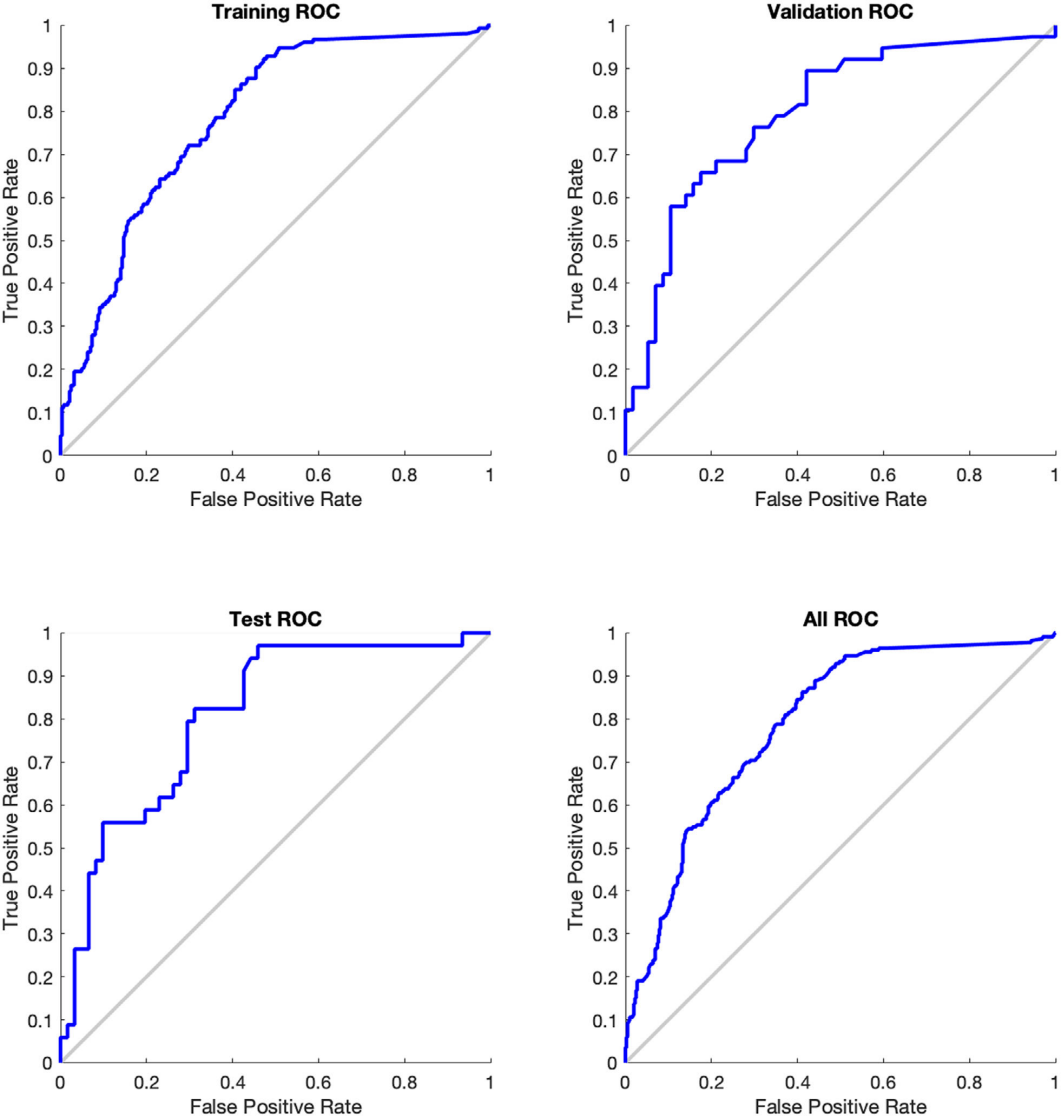
Receiver operating characteristic (ROC) curves demonstrating true positive rates for training, validation, and testing of the final model. All ROC includes the combined characteristics of the training, validation, and testing parameters.

The proposed learning model with the above parameters demonstrated 84%, 89.5%, and 90% specificity on the training, validation, and test sets, respectively (Table 2 and Fig. 5). Sensitivities were 55%, 50%, and 56%, respectively. The model with baseline and UPDRS‐II at 2 months showed lower sensitivity and specificity (48% and 77%, respectively). Models with more months of UPDRS‐II data generally improved in sensitivity and specificity but were comparable to the proposed model selected (Table 3).

**Table 2:** Confusion matrices for training and test sets.

Training set			
		Actual	
		Worsen	Same/Better
Predicted	Worsen	85 (19.3%)	239 (54.3%)
	Same/Better	69 (17.5%)	47 (10%)

Validation set			
		Actual	
		Worsen	Same/Better
Predicted	Worsen	19 (20%)	51 (54%)
	Same/Better	19 (20)	6 (6%)

Test set			
		Actual	
		Worsen	Same/better
Predicted	Worsen	19 (20%)	6 (6.3%)
	Same/Better	15 (15.8%)	55 (57.9%)

**Table 3:** Sensitivities and specificities for different learning models, incorporating scores every 2 months including baseline, up until the indicated month––demonstrating improvement as months used to predict increased.

Test characteristics	Months included				
	2	4	6	8	10
Sensitivity (%)	48	56	39	69	70
Specificity (%)	77	90	91	93	93
Positive likelihood ratio	2.12	5.68	4.39	10.2	10.2
Negative likelihood ratio	0.67	0.49	0.67	0.33	0.32
Positive predictive value (%)	48	76	64	86	87
Negative predictive value (%)	77	79	78	83	83
Accuracy (%)	68	78	76	84	84

## Discussion

A machine learning model, given an initial UPDRS‐II score, a score at 2 months, and a score at 4 months, could predict with high specificity, whether a patient would clinically worsen at 12‐month follow‐up. In an analysis of different approaches and parameters, this model exhibited highest sensitivity and specificity for the training, test, and validation divisions of data. In further tests, the model continued to improve––as data from more months were added as inputs into the neural network. To our knowledge, this is the first study to use only UPDRS‐II scores to predict clinical changes in QoL.

### Clinical impact of UPDRS‐II and machine learning

The UPDRS‐II score is a self‐reported questionnaire. This noninvasive, short, and convenient tool can be quickly and easily administered to gauge a PD patient's likelihood of progression. Noninvasive measures are both cost‐effective and convenient. With a high specificity prediction, this model can be used as a convenient screening measure for patients and providers. While it still remains difficult to predict which patients will significantly worsen over time, it is possible to *rule in* patients who may require additional attention and testing. Compared to traditional linear regression models, the proposed model demonstrates high clinical value. Traditional models have varied predictive capacity––with a range explaining around 21.7% to 58% of variance in QoL.^33^ At best model prediction using traditional linear regression analyses is insufficient and needs to be improved. Using solely UPDRS‐II scores and an ML‐based approach to determine those who will not clinically worsen at 12 months is a powerful tool in its own right––whose clinical use is only in early stages. Potentially, by using these tools in conjunction with traditional regression approaches, relevant contributors to QoL changes will become more apparent––improving the predictive capability of both models. Overall, however, this tool showed significant improvement over current linear regression techniques through 90% specificity and 56% sensitivity.

### Feasibility of predicting QoL changes with machine learning

While the model exhibited low sensitivity on training and test parameters, it did exhibit high specificity. The high specificity of the model indicates that it can be used to easily highlight PD patients who are not categorized as “staying the same/ better”. As the number of months used to predict change in UPDRS‐II at 12 months increased, the model improved. These improvements are consistent with other studies^29^ which indicate that predicting the course of clinically significant worsening in PD patients is difficult. In addition, the high specificity of the proposed model indicates that a low number of false negatives will be provided. Importantly, the positive predictive value (PPV) of the model was 76% and the negative predictive value (NPV) was 79%. Given a reasonable NPV, providers can be reasonably confident if a patient is classified as not significantly worsening. Physicians must still be cautious when evaluating patients classified as not worsening. However, this high PPV and NPV represents an important milestone in being able to feasibly predict progression of PD using a carefully designed machine learning algorithm.

### ML tools and model generalizability

As ML techniques become more commonly applied to medicine, it is increasingly important to understand how tools were developed and how those development techniques can be generalized to future models. In the case of our algorithm, we initiated the development process with various divisions of data––based on unique combinations of training, testing, and validation sets. Subsequently, within each of those divisions, we examined model parameters such as accuracy, positive predictive power, and sensitivity using variations in the number of input variables used as predictors. The combination of unique divisions of data, coupled with varying numbers of predictive variables is essential to optimize development––as both factors play major roles in overall model parameters. Here, as will be relevant for future models, there exists a balance between the number of input variables and model accuracy; for instance, a model that uses a large number of input variables may demonstrate high overall accuracy––however obtaining those variables may be challenging and impractical. Consequentially, one must balance real‐world challenges in acquiring actual data when considering model development as well.

### Model expansion and future directions

Given more data, it is quite probable that the model will continue to improve in its accuracy.^30^ While the PPMI database can be considered large, the number of patients included in this study were insufficient for a comprehensive and fully generalizable learning model. This study was limited by the number of available data; this can be overcome by more robust data. Our study was also limited by the retrospective nature of its design; future studies can evaluate predictions in change of QoL prospectively on a test set. Furthermore, it has been shown that different clinical subtypes of PD affect progression.^31^ In the absence of data on specific PD subtypes, the model was limited. Yet, the results obtained through the model are all the more remarkable given these limitations.

Variables such as MRI imaging data, along with more specific clinical and demographic variables––including age, gender, among others––could improve model sensitivity and specificity. It is important to note, however, that MRI and other sorts of imaging data may add multimodal complexity to the model and significantly increase time required for prediction.^32^, ^33^

One of the strengths of the model was the ease with which data could be collected and used; a simple survey in the form of UPDRS‐II was able to demonstrate high specificity of prediction. While using other features such as MRI and other sorts of biochemical markers could be useful, they may come at the cost of slowing down prediction. It will be important to take into consideration, not only the quantity and quality of data, but also the ease with which those data are collected and then processed for making predictions. Future work can utilize larger datasets, and potentially different parameters coupled with UPDRS‐II, to increase both sensitivity and specificity of a machine learning model. Furthermore, future studies can look at predicting changes in UPDRS‐II further out than 1 year. Given that there is still much unknown about PD, being able to predict changes in QoL with greater sensitivity and specificity––even further out––can add immense clinical benefit.

## Author Contributions

All authors contributed to the research project conception, organization, and execution. All authors contributed to design, review, and critique of the manuscript. TA contributed to the statistical analysis, manuscript drafting, as well as manuscript preparation.

## Conflict of Interest

Each author has no conflict of interest to disclose.

## Ethics Approval

This study was performed on a publicly available database and did not require IRB approval.
